# Case Report: Intravenous transplantation of autologous adipose tissue-derived mesenchymal stem cells attenuated atopic dermatitis

**DOI:** 10.3389/fmed.2026.1831398

**Published:** 2026-05-04

**Authors:** Kosuke Mabuchi, Yoshie Takahashi, Yuka Okinaka, Kana Nagase, Yosky Kataoka, Akihiko Taguchi

**Affiliations:** 1 Kanda Clinic, Tokyo, Japan; 2 BestCell Corporation, Osaka, Japan; 3 Department of Regenerative Medicine Research, Institute of Biomedical Research and Innovation at Kobe, Kobe, Japan; 4 Graduate School of Science, Technology and Innovation, Kobe University, Kobe, Japan

**Keywords:** adipose tissue, asthma, atopic dermatitis, gap junction, mesenchymal stem cell

## Abstract

Mesenchymal stem cells (MSC) transplantation had been shown to have a potential for reducing chronic pain. MSC transplantation suppresses excessive inflammation, and one of the potential mechanisms of action is a gap junction-meditated reduction of vascular permeability. Herein, we present the case of a 32-year-old female patient who underwent autologous adipose tissue-derived MSC transplantation for chronic pain and showed significant improvement in comorbid atopic dermatitis. Moreover, asthma attacks disappeared after MSC transplantation. This case shows that MSC therapy could be effective for treating allergic diseases, at least in responders. These findings encourage future studies to identify responders, surrogate markers, and the detailed therapeutic mechanisms of MSC transplantation for allergic diseases.

## Introduction

Treatments using mesenchymal stem cells (MSC) are initiated for various diseases (1) and MSC transplantation had been shown to have a potential for reducing chronic pain (2). MSC are known to suppress excessive inflammation (3), and one of the potential mechanisms of action is a gap junction-meditated suppression of activated endothelium and white blood cell that results in reduction of vascular permeability and tissue inflammation (4). A combination of activated vasculature, immune dysregulation, and epidermal barrier dysfunction has been proposed as the basic pathology of atopic dermatitis, although the pathological process is not fully understood and the cause varies between patients (5–7). Here, we present the case of a female patient who underwent autologous adipose tissue-derived MSC transplantation for chronic pain and showed significant improvement in comorbid atopic dermatitis.

## Case description

A 32-year-old woman with chronic lower back pain underwent intravenous autologous adipose tissue-derived MSC transplantation as described previously (2). Briefly, approximately 1 g of adipose tissue was obtained from the abdominal subcutaneous fat under local anesthesia and MSC were cultured in KBM ADSC-1 medium (Kohjin Bio, Saitama, Japan) for up to eight passages. These cells were frozen in freeze-damage protection fluid (Cellbanker; Zenogen Pharma, Fukushima, Japan) for multiple transplantations. In each transplantation, 1 × 10^8^ cells were intravenously injected over 30 min. After the first MSC transplantation, the patient experienced mild relief from lower back pain and underwent additional MSC transplantations 6 and 13 months later.

**Figure 1:**
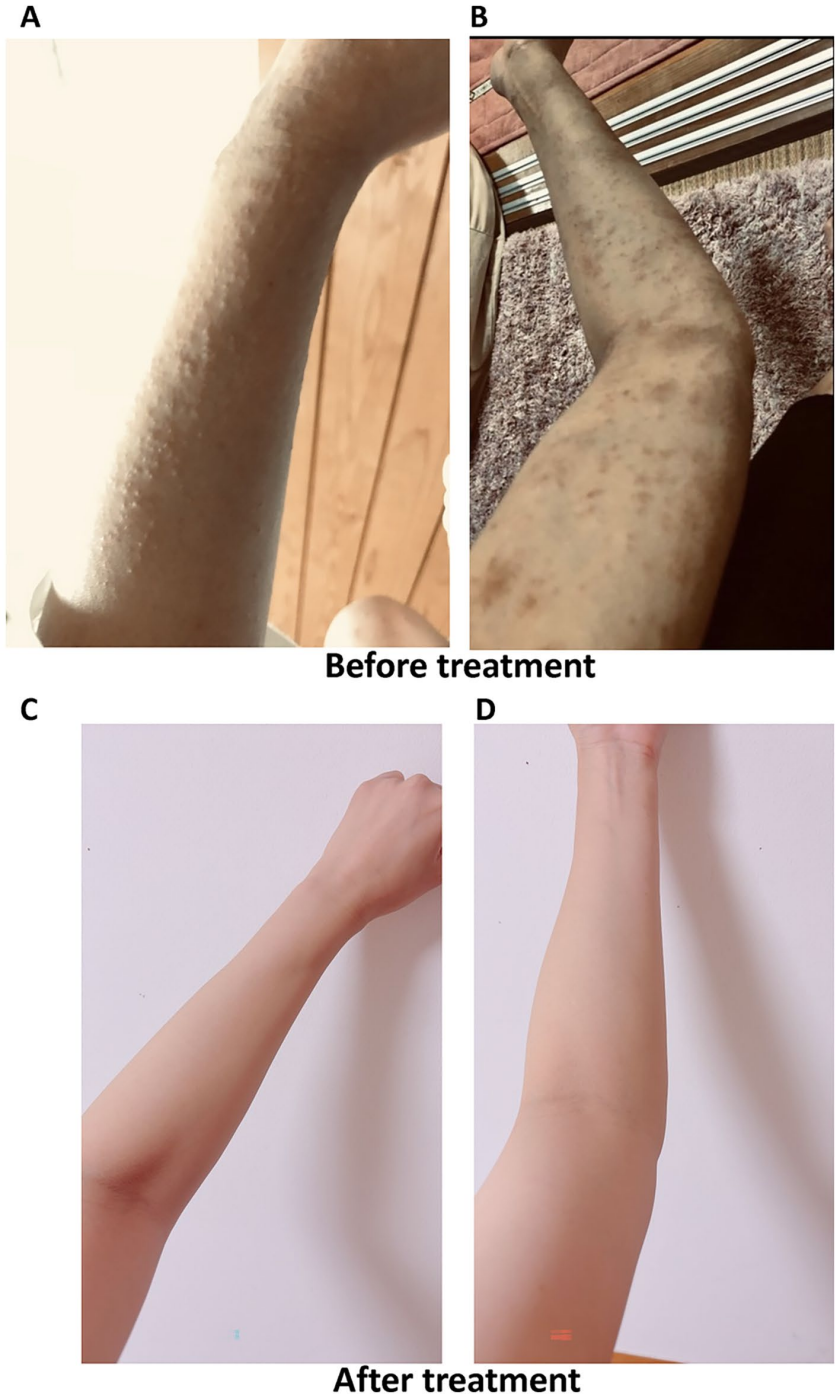
Atopic dermatitis on the arm before and after MSC therapy. **(A,B)** Patient had suffered from atopic dermatitis routinely **(A)** and mental stress or drinking alcohol worsened the symptoms **(B)**. **(C,D)** Atopic dermatitis has completely disappeared after three MSC transplantations. The patient no longer needs to use steroid cream and no atopic dermatitis appears even after drinking. MSC, mesenchymal stem cell.

This patient also had atopic dermatitis and experienced a vicious cycle of chronic itching, scratching, and worsening itching over the years (Figures 1A,B). Alcohol consumption was a clear exacerbating factor for her atopic dermatitis. Despite using a standard treatment for atopic dermatitis, including the application of 0.1% dexamethasone propionate, for the past 10 years, no suppression of atopic dermatitis was observed. On the night of the first MSC transplantation, she felt relief from chronic itching and did not need to scratch. Her atopic dermatitis symptoms improved after the second and third treatments, resulting in the disappearance of symptoms (Figures 1C,D), and the patient no longer needed to use steroid cream even after drinking alcohol. She also had asthma attacks about 5 to 6 times per year, but the attacks disappeared after three MSC injections. The frequency of the use of inhaled steroid decreased from 10 times per year to zero and the score of Asthma Control Test (8) had improved from 19 to 23. The patient was followed up for 6 months after third injection and no recurrence of atopic dermatitis nor asthma attack was observed.

**Figure 2:**
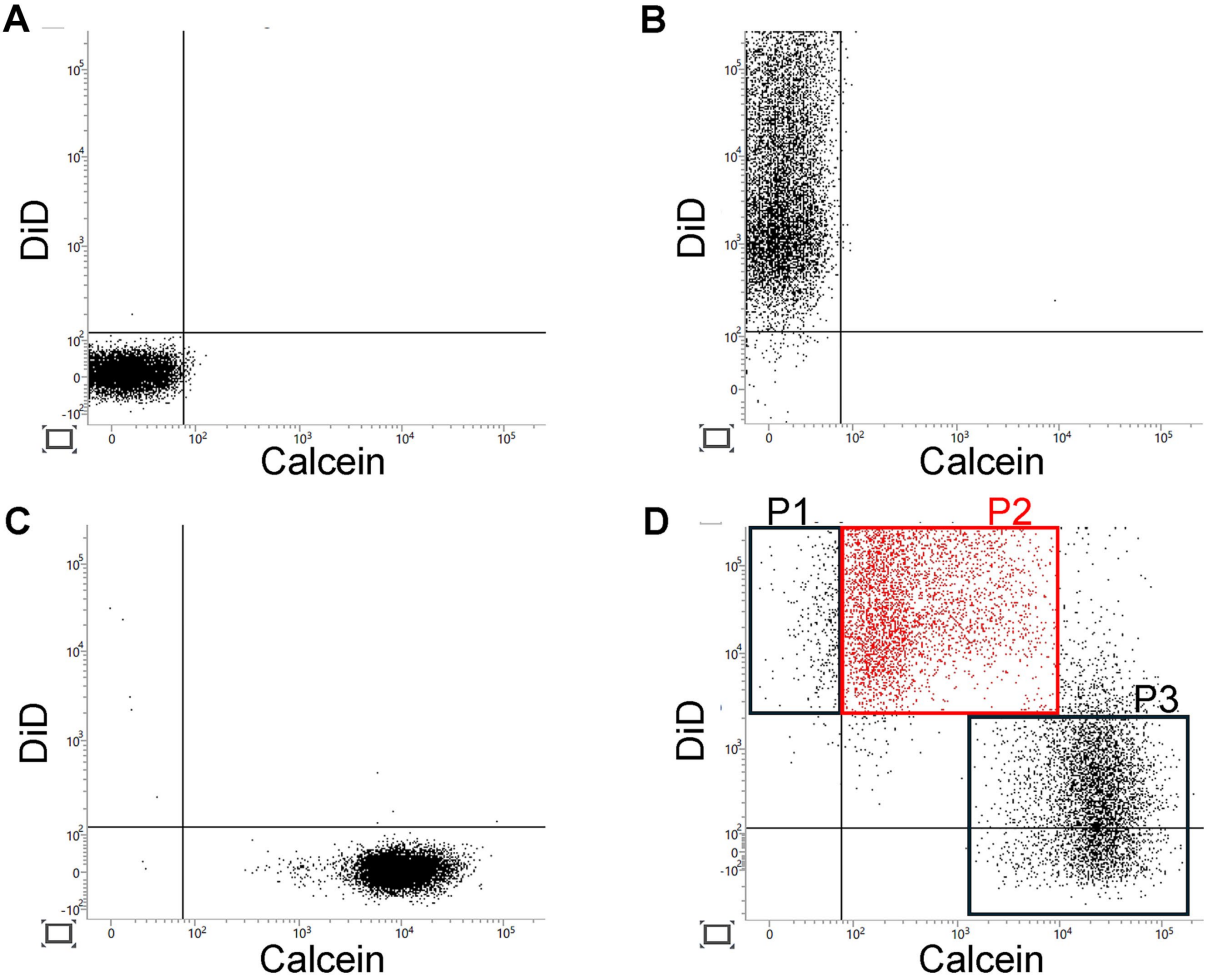
FACS analysis of calcein transfer potential of transplanted MSC. Results of FACS analysis of non-stained **(A)** cells, cells with red fluorescent (DiD) labeling of the membrane **(B)**, and cells with small water-soluble green fluorescent (calcein) loaded in the cytoplasm **(C)**. After co-culture of DiD-labeled and calcein-loaded cells, more than 90% of DiD-labeled cells became calcein-positive **(D)**. P1: DiD-labeled cells without calcein signal; P2: DiD-labeled cells with calcein signal; and P3: calcein-loaded cells. The percentage of MSC with the potential to remove small water-soluble molecules from other cells via gap junctions was calculated as follows: [number of calcein- and DiD-positive cells (P2)]/[number of total DiD-positive cells (P1 + P2)] × 100. MSC, mesenchymal stem cell.

MSC transplantation did not affect the complete blood count (Table 1). Serum level of stromal cell-derived factor 1 (SDF1) and vascular endothelial growth factor (VEGF) were measured before and 1 h after the third MSC transplantation using a Bio-Plex Multiplex System (Bio-Rad, Hercules, CA, United States) according to the manufacturer’s protocol. Compared with those before treatment, SDF1 and VEGF levels increased by approximately 30% 1 h after MSC transplantation (Table 2). The potential for gap junction-mediated small-molecule transfer by MSC was evaluated as described previously (2). More than 90% of transplanted MSC had the potential to remove small water-soluble substances from other cells via gap junctions (Figure 2).

**Table 1:** Complete blood count.

Blood count	Before 1st transplantation	Before 2nd transplantation	Before 3rd transplantation
RBC (×10^4^ cell/μL)	388	405	404
Platelet (×10^4^ cell/μL)	32.7	34.2	29.0
WBC (cells/μL)	6,900	6,900	6,600
WBC differential (%)			
Neutrophils	53.3	50.2	50.3
Lymphocytes	31.1	33.6	30.6
Monocytes	8.1	8.1	7.3
Eosinophils	6.8	7.0	11.1
Basophils	0.7	1.1	0.7

**Table 2:** Serum SDF1 and VEGF level.

Cytokine	Before 3rd transplantation	1 h after transplantation	% increase
SDF1 (pg/mL)	287.3	381.7	32.9
VEGF (pg/mL)	25.7	32.2	25.5

## Discussion

In this report, we present a case of atopic dermatitis improvement after intravenous autologous adipose tissue-derived MSC transplantation.

A clinical trial showed that allogeneic umbilical cord blood-derived MSC subcutaneous transplantation improves atopic dermatitis (9). In the clinical trial, approximately 40% enrolled patients achieved a 50% improvement in their Eczema Area and Severity Index score in 12 weeks. A significant decrease in the blood eosinophil percentage was observed 8 and 12 weeks after MSC transplantation, although no significant difference was observed 2 and 4 weeks after transplantation. Finally, in a clinical trial of allogeneic umbilical cord-derived MSC transplantation for asthma, the first patient showed 70 and 90% reductions in nebulizer and rescue inhaler usage, respectively, after MSC transplantation (10). In this case, no clear change in blood eosinophil percentage was observed after MSC transplantation, although autologous MSC transplantation significantly improved atopic dermatitis and asthma. Serum SDF1 and VEGF levels increased by approximately 30% 1 h after MSC transplantation. In patients with atopic dermatitis, increased serum levels of SDF1 had been reported (11). SDF1 is a chemokine that attracts white blood cells to inflammatory areas and damaged tissues, and MSC are the main source of SDF1 (12). The rapid increase in systemic serum SDF1 levels after MSC transplantation observed in this case might have exceeded the local, atopic dermatitis-induced SDF1 levels, thereby decreasing white blood cell infiltration to local dermatitis areas. Increased serum VEGF levels have been reported in patients with Alzheimer’s disease (13). VEGF is a key regulator of blood vessel growth and high levels of VEGF have been detected in the skin tissue of patients with atopic dermatitis (14). Although the correlation between increased VEGF levels after MSC transplantation and attenuated atopic dermatitis is unclear, MSC have the potential to increase serum VEGF levels by suppressing VEGF uptake into endothelial cells, resulting in reduced inflammation in damaged tissues (4). These findings indicate the necessity to identify markers for responders, together with surrogate markers of treatment, in future studies.

One of the potential mechanisms of action of MSC therapy is the gap junction-mediated suppression of activated endothelial and white blood cells, which reduces vascular permeability and tissue inflammation (4). In our previous report, we showed that approximately 80% of MSC have the potential to remove small molecules from other cells via gap junctions (2). In this case, more than 90% of transplanted MSC had this potential, and MSC transplantation improved atopic dermatitis and asthma. These findings encourage future studies that link the potential gap junction-mediated properties of MSC to the therapeutic effects of MSC transplantation in various allergic diseases.

This case report has limitations. Further pilot studies and subsequent prospective randomized trials are required to clarify the efficacy of MSC transplantation for atopic dermatitis and asthma. Furthermore, comparisons between autologous and allogenic MSC transplantation, identification of responders and surrogate markers, evaluation of the link between therapeutic effects and the potential gap junction-mediated properties of MSC, and elucidation of the detailed therapeutic mechanisms of MSC transplantation are required to establish MSC therapy as a general treatment for allergic diseases.

## Conclusion

This case demonstrates that MSC transplantation may have significant therapeutic potential for atopic dermatitis and asthma, at least in responders. The results of the blood tests indicated that changes in serum SDF1/VEGF levels can be used as a surrogate marker of MSC therapy. This case encourages future studies to establish novel treatments for various allergic diseases by elucidating the detailed mechanisms underlying MSC transplantation.

## Data Availability

The original contributions presented in the study are included in the article/supplementary material, further inquiries can be directed to the corresponding author.
